# First-line treatment and survival of newly diagnosed primary plasma cell leukemia patients in the Netherlands: a population-based study, 1989-2018

**DOI:** 10.1038/s41408-021-00415-5

**Published:** 2021-02-04

**Authors:** Mirian Brink, Otto Visser, Sonja Zweegman, Pieter Sonneveld, Annemiek Broyl, Niels W.C.J. van de Donk, Avinash G. Dinmohamed

**Affiliations:** 1 Department of Research and Development, Netherlands Comprehensive Cancer Organisation (IKNL), Utrecht, The Netherlands; 2 Department of Registration, Netherlands Comprehensive Cancer Organisation (IKNL), Utrecht, The Netherlands; 3 Amsterdam UMC, Vrije Universiteit Amsterdam, Department of Hematology, Cancer Center Amsterdam, Amsterdam, The Netherlands; 4 Department of Hematology, Erasmus MC Cancer Institute, Rotterdam, The Netherlands; 5 Department of Public Health, Erasmus University Medical Center, Rotterdam, The Netherlands

**Keywords:** Health care, Cancer

Dear Editor,

Primary plasma cell leukemia (pPCL) is the rarest and most aggressive entity within the spectrum of plasma cell dyscrasias, accounting for nearly 2% of all plasma cell dyscrasias^1^. Outcomes in pPCL have generally been grim with a median overall survival (OS) of <12 months^2–4^. Although currently there is a paucity of data on outcome in pPCL—which are mostly derived from retrospective studies—these data provided hints that the introduction of autologous stem cell transplantation (autoSCT) as well as proteasome-inhibitor (PI)-based and immunomodulatory (IMID)-based therapies have benefits for patients with pPCL^5–8^. Indeed, the most far-reaching population-based study—which included 445 pPCL patients diagnosed in the US during 1973–2009—demonstrated that survival in pPCL improved notably since 2006, especially for elderly patients^4^. This improvement was likely a consequence of the availability of PI-based and IMiD-based therapies for first-line treatment in the US from 2006 onwards. However, the improvement could not be directly linked to changing treatment practices, as information on treatment lacked in that study.

Given the scarcity of clinical and population-based studies in pPCL, we conducted a population-based study to assess trends in first-line therapy and survival among patients with pPCL diagnosed during a 30-year period in the Netherlands.

We identified all pPCL patients diagnosed between 1989 and 2018 from the nationwide Netherlands Cancer Registry (NCR), which was founded in 1989^9,10^. The primary endpoint was OS, defined as the time from diagnosis until death from any cause. Patients alive were censored on February 1, 2020. The log-rank test was used to test for differences between survival distributions. Multivariable Cox regression was applied to estimate the adjusted risk of mortality. Information on primary therapy was available for each patient in the NCR. Calendar period analyses (1989–2000, 2001–2007, and 2008–2018) were conducted to assess trends in primary therapy and OS according to age at diagnosis (≤65 and ≥66 years). The calendar periods were defined accordingly to changing treatment practices in multiple myeloma (MM) in the Netherlands. The full methods of our study are provided in the Supplemental. The Privacy Review Board of the NCR approved the use of anonymous data for this study.

The baseline characteristics of 226 patients with pPCL (median age, 66 years; range, 34–91 years; 52% males) included in this study are presented in Supplemental Table 1. One patient diagnosed at autopsy was excluded.

As shown in Fig. 1A, first-line treatment without SCT was commonly applied among patients aged ≤65 years in the periods 1989–2000 (65%) and 2001–2007 (59%). Thereafter, the incorporation of SCT into the first-line treatment increased markedly, namely from 23 to 60% between 1989–2000 and 2008–2018 (*P* = 0.004). As for patients aged ≥66 years, the proportion of patients who did not start with treatment was higher as compared to patients aged ≤65 years (*P* < 0.001). Although the proportion of patients aged ≥66 years not receiving therapy is decreasing over time, a substantial number of these patients still did not receive any therapy in the most recent calendar period (28% and 49% in 2008–2018 and 1989–2007, respectively; *P* = 0.030). However, noteworthy is the application of SCT among patients aged ≥66 in the most recent calendar period (8%; median age, 68 years; range, 66–69 years).

**Fig. 1:**
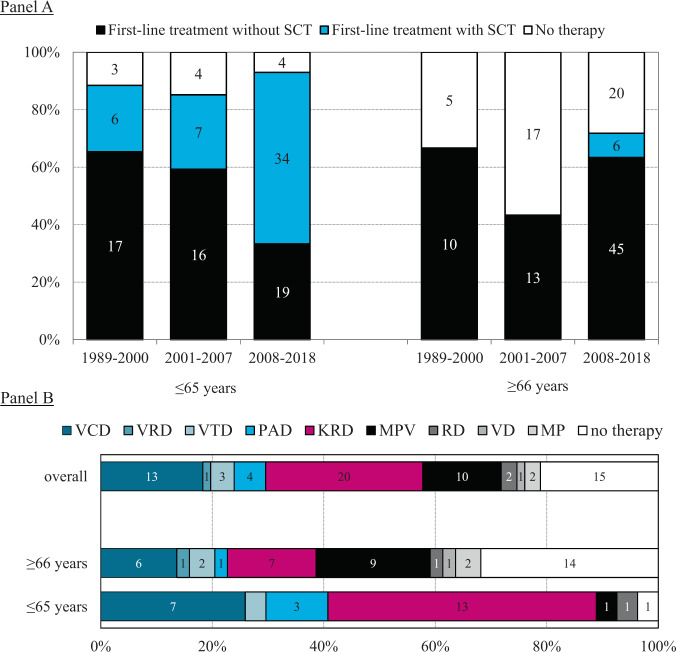
First-line treatment of patients with pPCL in the Netherlands. The absolute numbers of patients are depicted in the bars. **A** The first-line treatment for patients diagnosed with pPCL between 1989 and 2018 according to age at diagnosis and calendar period of diagnosis. **B** The information on the exact therapeutic regimen for diagnosed patients with pPCL between 2014 and 2018 according to age at diagnosis. Of note, patients who received KRD were treated within the setting of a clinical trial. pPCL primary plasma cell leukemia, SCT stem cell transplantation, bortezomib-dexamethasone (VD), VD with cyclophosphamide (VCD), VD with lenalidomide (VRD), VD with thalidomide (VTD), lenalidomide-dexamethasone (RD), RD with carfilzomib (KRD), VD with doxorubicin (PAD), melphalan-prednisone (MP), and MP with bortezomib (MPV).

Detailed data on baseline characteristics of 71 patients diagnosed in 2014–2018 are presented in Supplemental Table 2. Of these patients, the majority was treated with a PI-based regimen (58%; Fig. 1B). Twenty-two patients received an SCT as part of their first-line treatment, of whom nine received an autoSCT, eight the tandem of autoSCT and allogeneic SCT, and five a tandem autoSCT.

Overall, the median follow-up was 9.3 months (range, 0.03–198.3 months), whereas it was 52.0 months (range, 13.7–198.3 months) for patients alive at the end of follow-up. For all patients, median OS across the three calendar periods was 8.8, 5.0, and 14.4 months, respectively (*P* < 0.001; Supplemental Fig. 1). The corresponding estimates for patients aged ≤65 and ≥66 were 12.2, 13.8, and 28.4 months (*P* = 0.002), and 2.0, 2.4, and 6.4 months (*P* = 0.009), respectively (Fig. 2). Although OS improved over time, early mortality within 6 months after diagnosis remained high over time for both age groups. Among patients aged ≤65, the proportion of early mortality was 31%, 33%, and 25% for the three consecutive calendar periods (*P* = 0.667). The corresponding proportions for patients aged ≥66 years were 73%, 70%, and 49% (*P* = 0.067). The projected OS at 6 months and 1, 2, and 5 years post-diagnosis according to age at diagnosis and calendar period of diagnosis are presented in Fig. 2.

**Fig. 2:**
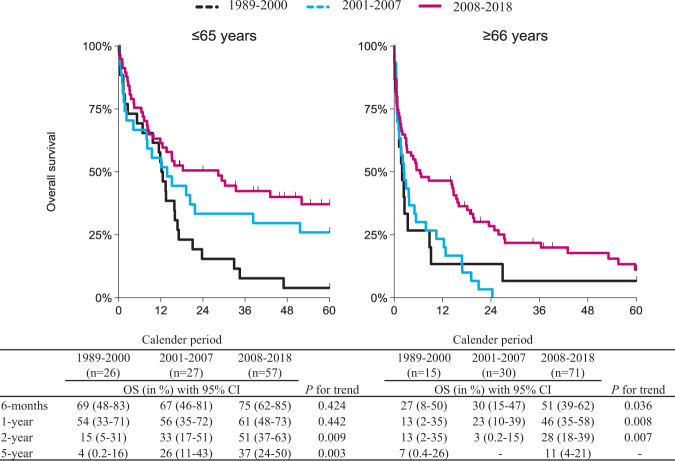
Overall survival (OS) of patients with pPCL in the Netherlands, 1989–2018. OS is stratified by calendar period of diagnosis and shown for the following age categories: ≤65 years (left panel), and ≥66 years (right panel). The *P*-value of the log-rank test for trend is indicated in the Figure.

When simultaneously adjusted for sex, age at diagnosis, and calendar period of diagnosis, patients diagnosed during 2008–2018 had a lower risk of mortality (adjusted hazard ratio [HR], 0.59; 95% confidence interval [CI] 0.42–0.84; *P* = 0.003), as compared to patients diagnosed before 2007. Furthermore, patients aged ≥66 years had a higher risk of mortality (adjusted HR, 2.19; 95% CI 1.63–2.94; *P* < 0.001), as compared to patients aged ≤65 years (Supplemental Table 3). However, when additionally adjusted for first-line treatment, the prognostic effects of calendar period and age lost statistical significance. This finding suggests that the broader application of therapy, especially the application of SCT, contributed to the improved survival over time, and this was irrespective of age (Supplemental Table 3).

In this comprehensive, nationwide, population-based study, the survival of pPCL patients improved over time. However, early mortality remains high, which is probably related to both disease-related and therapy-related complications. Our study is the first to directly link the improvements in survival with changes in first-line therapy over time. Collectively, patients with pPCL likely benefited from the therapeutic advances achieved over the past decades.

Our findings are mostly in keeping with a previous population-based study conducted in the US during 1973–2009^4^, and prospective and^10^ retrospective studies^11,12^, including a multicenter study conducted in Greece during 2000–2016^5^. However, in our study, the median OS of patients aged ≥66 years diagnosed during 2008–2018 was lower, as compared to the median OS of patients ≥66 years diagnosed during 2006–2009 in the US—possibly due to a delay in the introduction of more effective therapies in the Netherlands compared to the US. The importance of novel agent-based regimens in pPCL was also shown among 50 pPCL patients diagnosed in Greece during 2000–2016^5^. That study revealed that OS was significantly higher among patients managed with bortezomib-based regimens and autoSCT, as compared to those managed with more conventional therapies. Furthermore, our results compare less favorably with two relatively small prospective trials, which are the only trials published to date in pPCL^7,8^. These differences may be related to the use of novel agent-based regimens in all patients enrolled in these clinical trials, as compared to only 58% of the patients diagnosed in 2014–2018 in our study. Also, the selection of comparatively fit patients fulfilling the strict inclusion criteria of trials—as compared to the inclusion of all pPCL patients identified in population-based registries—may result in differences between the studies. Concerning stem cell transplantation, the OS in transplant-eligible pPCL patients in three large retrospective series^6,13,14^ was superior, as compared to the OS of pPCL patients aged ≤65 years diagnosed in 2014–2018 in our population-based study—of whom 63% received autoSCT. However, it is unknown which proportion of patients in the retrospective series^6,13,14^ was planned to undergo autoSCT, but eventually did not receive this treatment because of either early progression or early death. This particular selection bias may lead to an overestimation of the effectiveness of autoSCT in pPCL. Taken together, the therapeutic advances achieved in recent decades seem to gradually translate into tangible benefits for pPCL patients at the population level. Nevertheless, the specific design and conduct of prospective intervention studies in pPCL across different lines of therapy are imperative to advance the evidence-based management of pPCL. International collaboration is needed to accomplish such trials.

The main strength of our study includes the use of a nationwide population-based cancer registry with comprehensive data available on first-line treatment. Therefore, changing treatment practices over time could be assessed and directly linked to improvements in outcome. Limitations of our study mainly pertain to the lack of detailed information on first-line treatment throughout most of the study period (i.e., 1989–2013) and potential misclassification of pPCL as MM or vice versa, especially in earlier periods. Despite these limitations, cancer registries remain the standard for cancer surveillance activities.

In summary, the population-level survival of pPCL patients improved significantly over time. Notwithstanding this encouraging finding, survival in pPCL remains unsatisfactory, especially among the elderly. Therefore, the design and conduct of forthcoming prospective intervention studies for patients with pPCL are essential to establish evidence-based treatment recommendations, which, in turn, may further improve the outcome in this patient population.

## Supplementary information

Supplemental material
