# Supplementary material for: First-line treatment and survival of newly diagnosed primary plasma cell leukemia patients in the Netherlands: a population-based study, 1989-2018
Source: Blood Cancer J. 2021 Feb 4;11(2):22. doi: 10.1038/s41408-021-00415-5 (PMC7873037; doi:10.1038/s41408-021-00415-5)
Supplement: Supplementary file 1 — Supplemental material [file 41408_2021_415_MOESM1_ESM.docx]

**ONLINE APPENDIX**

**Title**

First-line treatment and survival of newly diagnosed primary plasma cell leukemia patients in the Netherlands: a population-based study, 1989-2018

Full list of authors and affiliations is available at the end of the correspondence.

**SUPPLEMENTAL METHODS**

**The Netherlands Cancer Registry**

Nationwide since 1989, the population-based Netherlands Cancer Registry (NCR), which is maintained and hosted by the Netherlands Comprehensive Cancer Organisation (IKNL), has a coverage of at least 95% of all malignancies in the Netherlands^1^. All newly diagnosed malignancies in the Netherlands are reported to the NCR via the Nationwide Network of Histopathology and Cytopathology, and the National Registry of Hospital Discharges (i.e. inpatient and outpatient discharges). Information on dates of birth and diagnosis, sex, disease topography and morphology, and first-line therapy is routinely recorded in the NCR by trained registrars of the NCR through retrospective medical records review. Topography and morphology are coded according to the International Classification of Diseases for Oncology (ICD-O), of which the morphology codes 9733 and 9830 were used for identifying patients with primary plasma cell leukemia (pPCL) in the current study. Information on the last known vital status for all patients (i.e. alive, death, or emigration) is obtained through annual linkage with the Nationwide Population Registries Network that holds vital statistics on all residents in the Netherlands.

Primary treatment within one year after pPCL diagnosis was defined as no therapy and therapy with or without stem cell transplantation (SCT). Detailed information on baseline charateristics and the exact therapeutic regimen and type of SCT was available in the NCR for patients diagnosed from 2014 onwards.

According to the Central Committee on Research involving Human Subjects (CCMO), this type of observational study does not require approval from an ethics committee in the Netherlands. The Privacy Review Board of the NCR approved the use of anonymous data for this study.

**Statistical analyses**

The Fisher’s exact test was applied to compare categorical variables. OS was defined as the time between pPCL diagnosis and death from any cause. Patients alive were censored at February 1, 2020. OS was calculated for three calendar periods (1989-2000, 2001-2007, 2008-2018) and two age categories (≤65 and ≥66 years). The calendar periods were based on the implementation of autoSCT (≥2001) and first-generation novel agents (≥2008) into treatment algorithms for patients with multiple myeloma (MM) in the Netherlands. Survival distributions were compared with the log-rank test. Multivariable evaluation of OS was performed using Cox regression, with adjustment for sex, age at diagnosis, calendar period of diagnosis, and primary therapy. A *P*<0.05 indicated statistical significance. All statistical analyses were performed with STATA Statistical Software Release 14.2 (College Station, TX, USA).

**Authors:** Mirian Brink,^1^ Otto Visser,^2^ Sonja Zweegman,^3^ Pieter Sonneveld,^4^ Annemiek Broyl,^4^ Niels W.C.J. van de Donk,^3*^ Avinash G. Dinmohamed^1,3,5*^

*These authors jointly supervised this work.

**Author details:** ^1^Department of Research and Development, Netherlands Comprehensive Cancer Organisation (IKNL), Utrecht, The Netherlands; ^2^Department of Registration, Netherlands Comprehensive Cancer Organisation (IKNL), Utrecht, The Netherlands; ^3^Amsterdam UMC, Vrije Universiteit Amsterdam, Department of Hematology, Cancer Center Amsterdam, Amsterdam, The Netherlands; ^4^Department of Hematology, Erasmus MC Cancer Institute, Rotterdam, The Netherlands; ^5^Department of Public Health, Erasmus University Medical Center, Rotterdam, The Netherlands

**Correspondence:** Mirian Brink, Department of Research and Development, Netherlands Comprehensive Cancer Organisation (IKNL), Godebaldkwartier 419, 3511 DT Utrecht, The Netherlands; e-mail: m.brink@iknl.nl.

**Supplemental references**

1. Schouten LJ, Hoppener P, van den Brandt PA, Knottnerus JA, Jager JJ. Completeness of cancer registration in Limburg, The Netherlands. *Int J Epidemiol*. 1993;22(3):369-376.

**Supplemental results**

**Supplemental figure 1.** Overall survival (OS) of patients with pPCL in the Netherlands, 1989-2018. OS is stratified by calendar period of diagnosis; *P*-value for log-rank test for trend.

*P*=0.0009

**Supplemental Table 1.** Baseline characteristics of patients diagnosed in 1989-2018

in the Netherlands, stratified by calendar period.

| **Characteristics** | **Calender period** | | | | | | **Total** | |
| --- | --- | --- | --- | --- | --- | --- | --- | --- |
|  | **1989-2000** | | **2001-2007** | | **2008-2018** | |  |  |
|  | **No.** | **(%)** | **No.** | **(%)** | **No.** | **(%)** | **No.** | **(%)** |
| **Total No. of patients** | 41 |  | 57 |  | 128 |  | 226 |  |
| **Sex** |  |  |  |  |  |  |  |  |
| Male | 25 | (61) | 27 | (47) | 65 | (51) | 117 | (52) |
| Female | 16 | (39) | 30 | (53) | 63 | (49) | 109 | (48) |
| **Age, years** |  |  |  |  |  |  |  |  |
| Median, IQR | 63 (51-71) | | 66 (57-76) | | 68 (61-75) | | 66 (59-74) | |
| ≤65 | 26 | (63) | 27 | (47) | 57 | (45) | 110 | (49) |
| ≥66 | 15 | (37) | 30 | (53) | 71 | (55) | 116 | (51) |
| **Follow-up, months** |  |  |  |  |  |  |  |  |
| Median | 8.8 | | 5.0 | | 14.0 | | 9.3 | |
| range | 0.03-85.3 | | 0.03-198.3 | | 0.03-119.9 | | 0.03-198.3 | |
| **Vital statistics** |  |  |  |  |  |  |  |  |
| Alive | - | - | 4 | (7) | 30 | (23) | 34 | (15) |
| Death | 41 | (100) | 53 | (93) | 98 | (77) | 192 | (85) |

Abbreviations: IQR; inter quartile range.

**Supplemental Table 2.** Baseline characteristics of pPCL patients diagnosed in 2014-2018 in the Netherlands

| **Characteristics** | **No.** | **(%)** |
| --- | --- | --- |
| **Total No. of patients** | 71 |  |
| **Sex** |  |  |
| Male | 39 | (55) |
| Female | 32 | (45) |
| **Age, years** |  |  |
| Median, IQR | 69 (63-77) | |
| ≤65 | 27 | (38) |
| ≥66 | 44 | (62) |
| **Follow-up, months** |  |  |
| Median | 13.9 | |
| range | 0.03-68.1 | |
| **Vital statistics** |  |  |
| Alive | 20 | (28) |
| Death | 51 | (72) |
| **≥60% BM Plasma cells** | 41 | (58) |
| **Thrombocytopenia^*^** | 33 | (47) |
| **Cytogenetic risk^1^** |  |  |
| Standard-risk | 18 | (25) |
| High-risk | 32 | (45) |
| Unknown | 21 | (30) |
| **ISS^2^** |  |  |
| 1 | 5 | (7) |
| 2 | 8 | (11) |
| 3 | 31 | (44) |
| Unknown | 27 | (38) |
| **CRAB criteria** |  |  |
| ≥1 | 64 | (90) |
|  |  |  |
| Hypercalcemia^3^ | 17 | (24) |
| Renal insufficiency^4^ | 17 | (24) |
| Anemia^5^ | 44 | (62) |
| 1 or more bone lesions | 40 | (56) |

Abbreviations: BM, bone marrow; IQR; inter quartile range, ISS; International Staging System, CRAB; Calcium Renal Anemia Bone laesions

^*^Thrombocytes <100*10^9^/L

^1^High-risk: presence of translocation (4;14), translocation (14;16) and/or deletion 17p; standard-risk: all other aberrations or no abnormalities.

^2^ISS stage 1: serum β2-microglobuline<3.50 mg/L& serum albumine>=35 g/L; ISS stage 3: serum β2-microglobuline >=5.50 mg/L; ISS stage 2; neither stage 1 nor 3; unknown: serum β2-microglobuline and/or serum albumin unknown.

^3^Serum calcium >2.75 mmol/L.

^4^Creatinine>177 mmol/L.

^5^Hemoglobin<6.2 mmol/L.

**Supplemental Table 3.** Results of the multivariable Cox regression.

|  | **Model w/o type of primary therapy** | | | **Model w/ type of primary therapy** | | |
| --- | --- | --- | --- | --- | --- | --- |
|  | **HR** | **95% CI** | **P** | **HR** | **95% CI** | **P** |
| **Period of diagnosis** |  |  |  |  |  |  |
| 1989-2000 | 1.24 | 0.82-1.87 | 0.311 | 1.50 | 0.98-2.30 | 0.062 |
| 2001-2007 | 1 | (ref) |  | 1 | (ref) |  |
| 2008-2018 | 0.59 | 0.42-0.84 | 0.003 | 1.09 | 0.75-1.58 | 0.663 |
| **Female sex** | 0.97 | 0.73-1.29 | 0.829 | 0.87 | 0.65-1.16 | 0.329 |
| **Age at diagnosis, years** |  |  |  |  |  |  |
| 18-65 | 1 | (ref) |  | 1 | (ref) |  |
| ≥66 | 2.19 | 1.63-2.94 | <0.001 | 1.27 | 0.92-1.77 | 0.150 |
| **Primary treatment** |  |  |  |  |  |  |
| Chemotherapy |  |  |  | 1 | (ref) |  |
| Chemotherapy+SCT |  |  |  | 0.35 | 0.22-0.55 | <0.001 |
| No therapy |  |  |  | 3.40 | 2.36-4.88 | <0.001 |

Abbreviations: HR, hazard ratio; CI, confidence interval; SCT, stem cell transplantation.
